# How ligands modulate the gastric H,K-ATPase activity and its inhibition by tegoprazan

**DOI:** 10.1016/j.jbc.2024.107986

**Published:** 2024-11-14

**Authors:** Nicole T. Cerf, Gerardo Zerbetto de Palma, Natalya U. Fedosova, Claudia V. Filomatori, Rolando C. Rossi, Santiago E. Faraj, Mónica R. Montes

**Affiliations:** 1 Consejo Nacional de Investigaciones Científicas y Técnicas (CONICET) – Universidad de Buenos Aires, Instituto de Química y Fisicoquímica Biológicas “Prof. Alejandro C. Paladini” (IQUIFIB), Buenos Aires, Argentina; 2 Department of Biomedicine, Aarhus University, Aarhus, Denmark

**Keywords:** P-type ATPase, inhibition mechanism, kinetic models, P-CABs, tegoprazan, H,K-ATPase, gastric acidity treatment

## Abstract

The introduction of potassium-competitive acid blockers (P-CABs) has been a major innovation in gastric H,K-ATPase inhibition and many laboratories are actively engaged in the development of novel molecules within this class. This work investigates the interaction between H,K-ATPase and tegoprazan, a representative of the P-CABs group, in terms of K^+^ and H^+^ binding, through functional and structural analyses. First, by studying the H,K-ATPase activity, we found a model to describe the non-Michaelis-Menten kinetics through a “ping-pong” mechanism that explains a stoichiometry of 1 H^+^, 1 K^+^, and 1 ATP molecule, but also considering the influence of H^+^ on the ionization states of the protein. A kinetic evaluation of the inhibition of tegoprazan denotes the binding to two different intermediates states with apparent K_d_ (μM) 0.56 ± 0.04 and 2.70 ± 0.24 at pH 7.2. Molecular dynamics simulations revealed important changes in the interactions of tegoprazan with the transmembrane residues depending on whether the site contains K^+^ or not. This explains the decrease in affinity as a function of K^+^ concentration observed in the kinetic experiments. On the other hand, the structures predict that the protonation of tegoprazan is responsible for the change in its dihedral angle. The rotation of the benzimidazole ring allows the inhibitor to be positioned further into the luminal cavity, a situation compatible with the higher inhibition affinity of H,K-ATPase measured at low pH. Results presented herein will provide a basis for the rational design of novel P-CABs ligands.

P-type cation pumps constitute an important family of transport ATPases, with a key role in cellular function. The gastric H,K-ATPase is responsible for the acidification of the stomach by pumping H^+^ ions in exchange for extracellular K^+^ ions. Whether the transport stoichiometry is 2H^+^/2K^+^/ATP or 1H^+^/1K^+^/ATP has long been debated (1, 2, 3, 4) although the analysis of the crystal structures revealed a single K^+^ bound to the cation-binding site (5). P-type ATPases are also known as E1-E2 pumps because their functional schemes include two major conformations of the protein, E1 and E2 states, with the alternating exposure of the cation-binding site to the intracellular and extracellular side of the membrane. The E1 conformation of the H,K-ATPase exhibits high affinity for H^+^, whereas E2 presents high affinity for K^+^.

Since, the H,K-ATPase is embedded in the plasma membrane of parietal cells, it is a perfect target for the drugs against acid-related disorders. Indeed, the preferent treatment for reducing gastric acid secretion is currently the selective inhibition of the gastric pump. During the latter part of the last century, the use of the “proton pump inhibitors” (PPIs) marked a significant advance in the pharmacological control of gastric acid secretion (6). Despite the high efficacy of PPIs, some pharmacological shortcomings have been identified, leading to major efforts to develop promising candidates, the “potassium competitive acid blockers” (P-CABs) (7). This new group of inhibitors prevents K^+^ binding to the H,K-ATPase and thus reduces H^+^ transport activity.

In contrast to PPIs, P-CABs do not require acid activation and their interactions with the H,K-ATPase are reversible. Representative of this group, tegoprazan ((S)-4-((5,7-difluorochroman-4-yl)oxy)-N,N,2-trimethyl−1Hbenzo[d]imidazole-6-carboxamide) has been approved for use in humans after successful demonstration of its *in vivo* efficacy in animal model (8), healthy subjects (9), and patients with gastro-esophageal reflux disease, gastric ulcer, or infected with *Helicobacter pylori* (10, 11). However, since certain P-CABs such as SCH28080 and Linaprazan have shown adverse effects in clinical trials, the design and testing of new analogs are ongoing (12, 13). While computational methods provide valuable information for rational drug design (14), functional validation is mandatory. Indeed, the analysis of H,K-ATPase inhibition and, particularly, how the physiological ligands, K^+^ and H^+^, influence the interactions of P-CABs will provide clues to unravel inhibitors binding requirements and improve ligands design.

Many studies on the catalytic mechanism of the H,K-ATPase have been devoted to evaluating partial reactions of the cycle (*e.g*., (15, 16)), and very few analyses have been carried out systematically to describe the overall mechanism of the enzyme. This is mainly due to the complexity of the P-type ATPase system and due to the difficulty of non-Michael-Menten kinetics analysis (17). This paper examines the global functioning of the H,K-ATPase and presents an adequate model to simulate the nonhyperbolic dependence of ATP hydrolysis on H^+^ and K^+^ as a basis for assessing tegoprazan inhibition. Although it has been widely debated whether proton movement through membrane proteins occurs by hydronium ion transfer or by proton jumping mechanism (18, 19, 20), here we show that the “pumping” mode, which is compatible with a ping-pong model, satisfactorily explains the exchange of one H^+^ for one K^+^ per cycle of the H,K-ATPase. Having described the H,K-ATPase catalytic cycle, a comprehensive kinetic characterization elucidates how K^+^ and H^+^ modulate the inhibition of the gastric pump by tegoprazan. We used computational simulations to propose the molecular-level interactions involved in that modulation.

## Results

### Interactions of K^+^ and H^+^ with the H,K-ATPase

**Figure 1:**
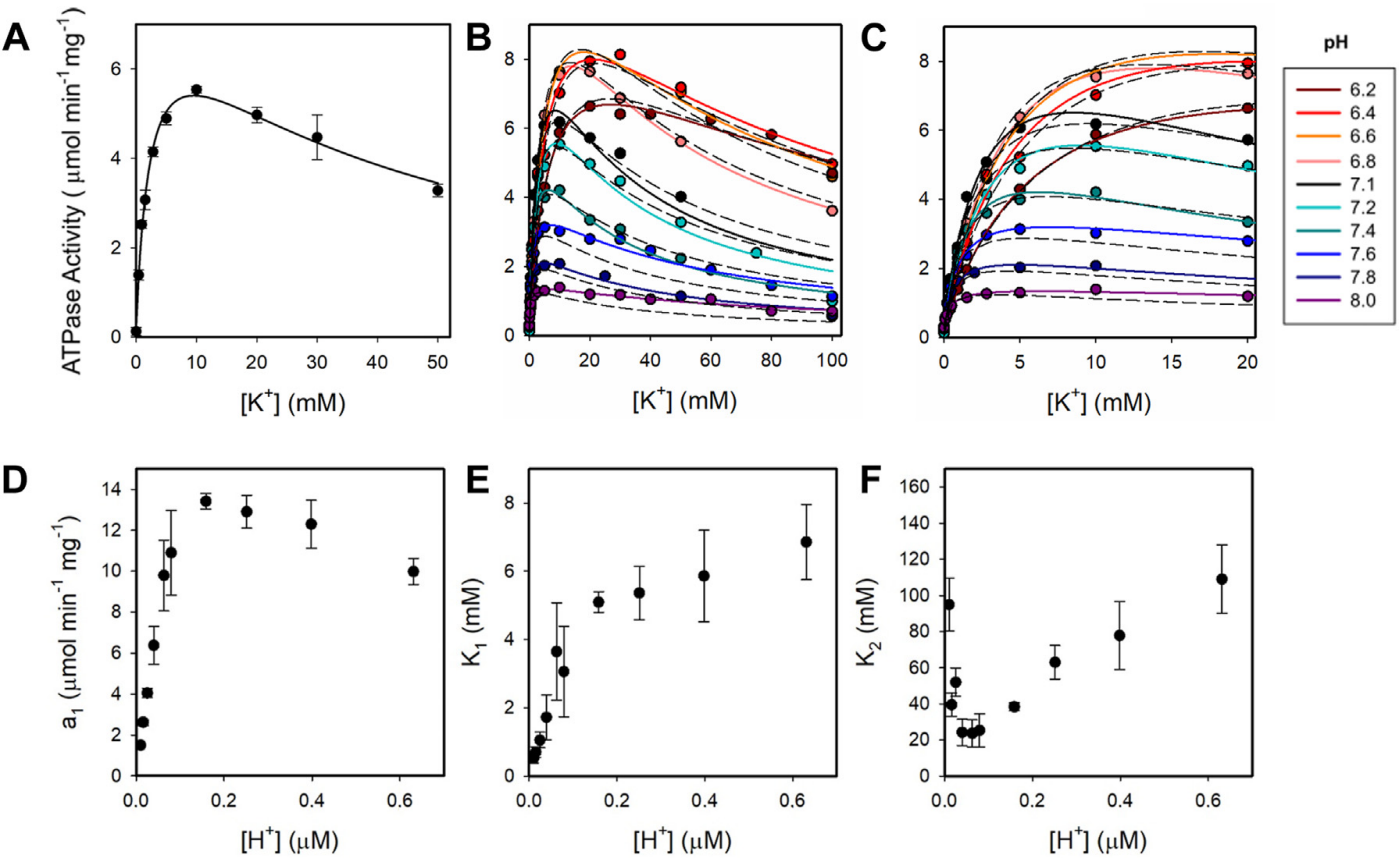
**Dependence of the H,K-ATPase on K**^**+**^**at different H**^**+**^**concentrations.***Panels A*–*C*, typical curves describing dependencies of ATPase activity. *Continuous lines* are the plot of Equation 1. Fitted values of the parameters $a_{1}$ (panel D), K_1_ (panel E), and K_2_ (panel F) from Equation 1 for the data show in Figure 1*B*. Reaction media contained 8 μg protein ml^−1^, 0.25 mM EDTA, 2 mM MgCl_2_, 2 mM ATP, and choline chloride to maintain constant ionic strength. Error bars, standard error. *Dashed lines* represent the best fitting of Equation 2, derived from the model in Figure 3, to the whole set of data for the parameters shown in Table 1.

We initially measured the ATPase activity as a function of [K^+^]. It can be observed (Fig. 1 panel *A*) an increase of the enzyme activity, followed by a decrease at higher [K^+^] (21). The results were analyzed using nonlinear regression with the following empirical equation:

(1) $$Act\left(\left[K^{+}\right]\right)=\frac{a_{0}+a_{1}\cdot \frac{\left[K^{+}\right]}{K_{1}}}{1+\frac{\left[K^{+}\right]}{K_{1}}+\frac{{\left[K^{+}\right]}^{2}}{K_{1}\cdot K_{2}}}$$

Equation 1 is a second-order rational function of [K^+^], $a_{0}$ and $a_{1}$ are coefficients with units of velocity, $a_{0}$ corresponds to the K^+^-independent ATPase activity, and $a_{1}$ accounts for the maximal ATPase activity that would be achieved in the absence of K^+^ inhibition. K_1_ and K_2_ are apparent dissociation constants expressed in millimolar, the former representing the high affinity binding of K^+^, which activates the enzyme cycle, whereas K_2_ reflects a low affinity binding of K^+^ that inhibits ATP hydrolysis. The second order in Equation 1 is consistent with the binding of K^+^ to at least two different intermediates during the catalytic cycle.

Now, we explored the dependence of H,K-ATPase activity with both substrates; measurements were performed for [K^+^] from 0 to 100 mM and [H^+^] from 0.001 to 0.631 μM (pH 8–6.2) (Fig. 1, *B* and *C*). Equation 1 was fitted for each H^+^ concentration, and the values of $a_{1}$, K_1_, and K_2_ are plotted as a function of [H^+^]. As H^+^ concentration increases, i) $a_{1}$ first rises and then decreases (panel D); ii) the apparent high affinity dissociation constant for K^+^, K_1_ increases (panel E); and iii) K_2_ first decreases and then increases (panel F).

**Figure 2:**
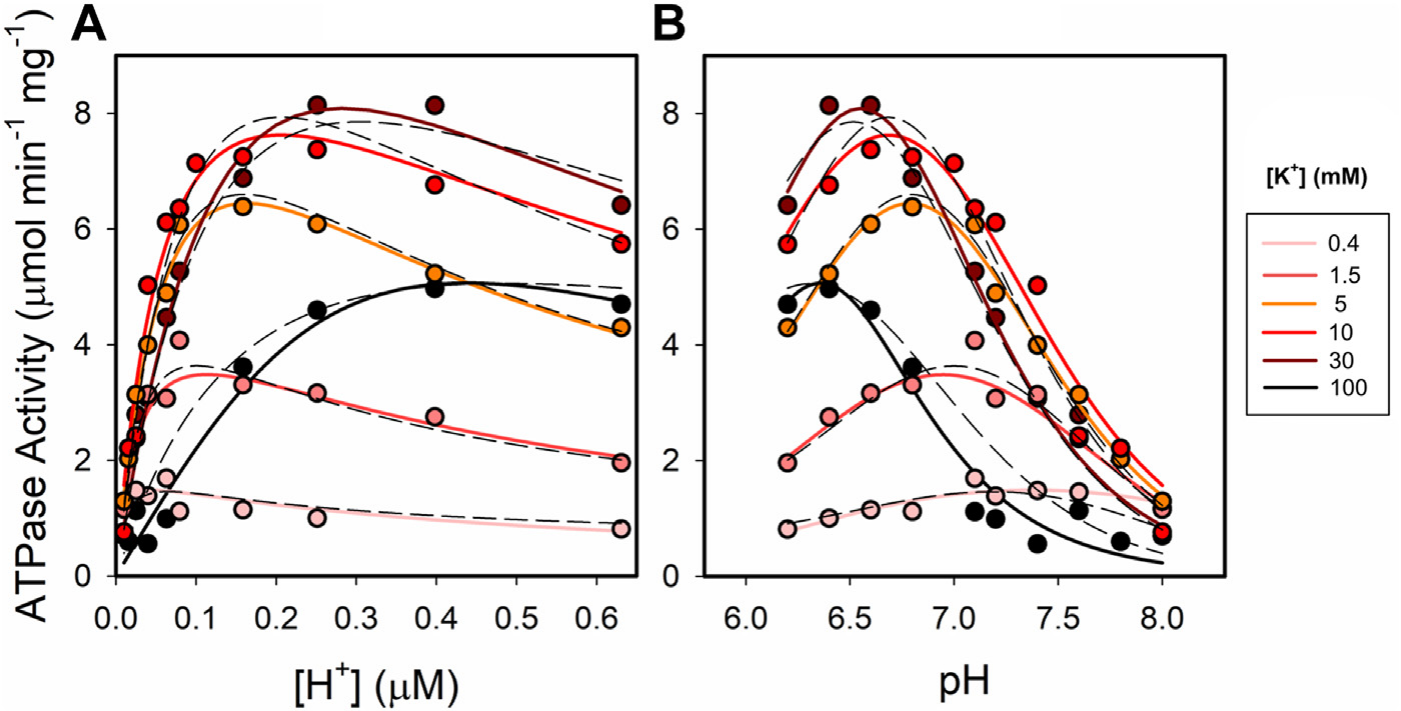
**Dependence of ATPase activity on [H**^**+**^**] at different [K**^**+**^**].** In panel A. the data from Figure 1 have been plotted as a function of [H^+^]. Panel B shows the bell-shaped profile representing the dependence of enzyme activity on pH. The *continuous lines*, included to guide the eye, represent an empirical equation for each cation concentration. *Dashed lines* represent the best fitting of Equation 2, derived from the model in Figure 3, to the whole set of data for the parameters shown in Table 1.

Results from Figure 1 were replotted in Figure 2 to inspect the dependence of ATPase activity on [H^+^]. It can be seen that, when [K^+^] rises, higher H^+^ concentrations are required to obtain half-maximal (apparent K_M_) and maximal activities. This is reflected in Figure 2*B* by the peaks becoming higher and shifting to the left as [K^+^] increases. The rise in apparent K_M_ and maximal velocity with cation concentration seen in Figures 1*C* and 2*A* is compatible with a ping pong mechanism for H^+^ and K^+^ (Fig. S1 under Supporting information). Figure 2 also reveals the inhibition caused at higher H^+^ and K^+^ concentration, which will be discussed below.

In studying the H,K-ATPase activity as a function of pH, we must though consider not only the effects of H^+^ as the physiologically transported cation but also as responsible for the protonation of titratable sites in the protein. It is well known that protonation/deprotonation of key amino acids could lead to nonactive enzymatic states, a condition also illustrated by the bell-shaped curves (Fig. 2*B*).

### A minimal model

**Figure 3:**
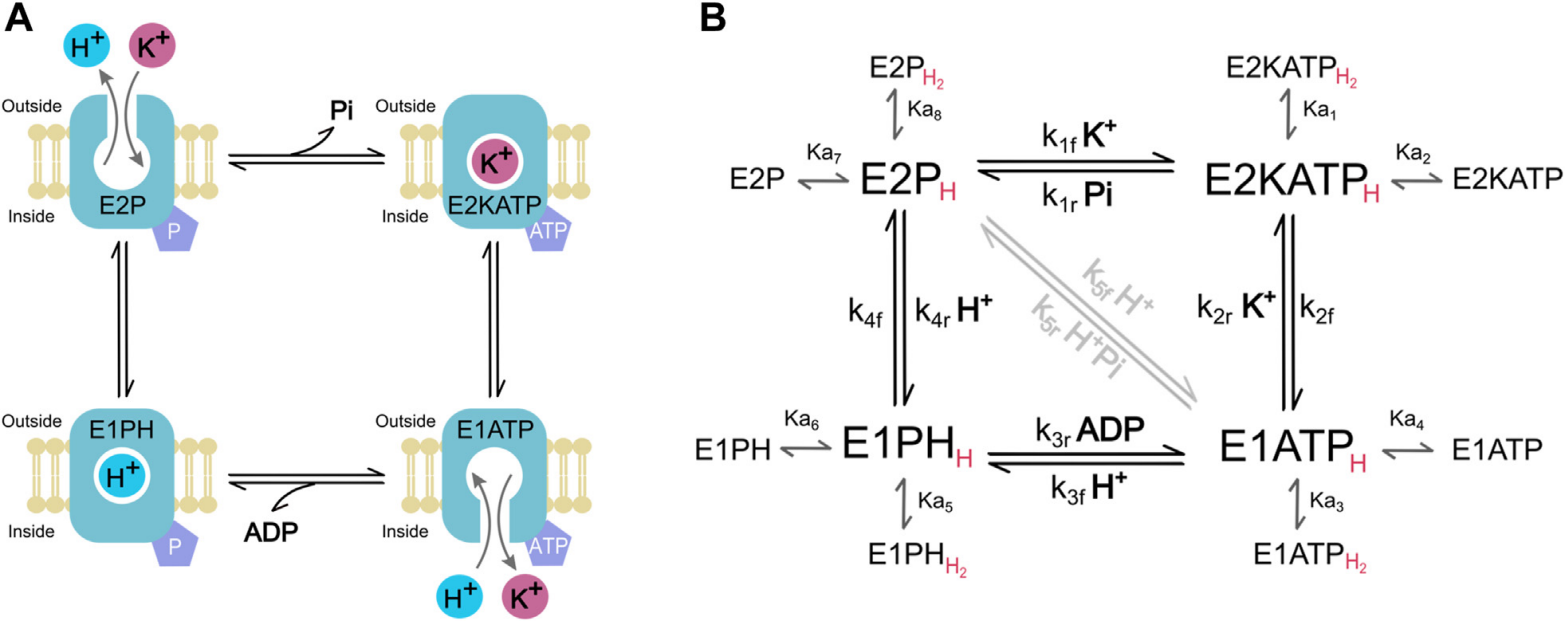
**A minimal model for H**^**+**^**, K**^**+**^**, and H,K-ATPase interactions.** Panel A shows K^+^ binding to E2P from outside the cell and its release to the cytoplasmic side from E1ATP. H^+^ is bound by E1ATP from inside the cell and is released to the extracellular side from E2P. In the scheme, cartooned for better comprehension, each enzyme intermediate is represented with its cytoplasmic side (“inside”) facing down. Panel B shows the catalytic cycle reproducing the results in a noncompartmentalized system: the intermediates E2P, E2KATP, E1ATP, and E1P, the rate constants k_i_'s, and the steps involved in cation binding. Every intermediate of the cycle is represented in equilibrium (K_ai's_) between the nonprotonated, monoprotonated, and diprotonated states (*red*, H). The pathway controlled by k_5_ has been colored *gray* because it is only relevant under nonphysiological conditions (absence of K^+^).

In light of the above results, we suggest the minimal scheme shown in Figure 3. It is based on the classical Albers-Post model that proposes four major conformations, phosphorylated and nonphosphorylated E1 and E2.

The model poses that K^+^ and H^+^ are transported by an alternating access mechanism between inward-facing E1 and outward-facing E2 conformations. This transforms a high-affinity ligand binding site on one side of the membrane into a low-affinity site on the other (22).

For an ATP hydrolysis cycle, the extracellular K^+^ and K^+^_e_, binds to E2P and promotes the enzyme dephosphorylation leading, at saturating ATP concentration, to E2KATP. Then, a conformational change from E2 to E1 releases K^+^_i_ toward the intracellular side. The binding of H^+^_i_ to the cytoplasmic access leads to enzyme phosphorylation and subsequently a new conformational change from E1 to E2, releases H^+^_e_ to the extracellular side (Fig. 3*A*). Since we are working with noncompartmentalized enzyme preparation (the same medium bathes the internal and external accesses), the concentration of a transported cation as substrate and as product are necessarily equal and cannot be changed independently (23) Hence, in our experimental conditions, [K^+^_e_] = [K^+^_i_] = [K^+^] and [H^+^_i_] = [H^+^_e_] = [H^+^], which implies that the products will always be present and can reverse the reaction steps that involve their release (Fig. 3*B*). The inhibition observed at high K^+^ concentrations in Figure 1, *A* and *B* can be explained by the hypothesis that K^+^ competes with H^+^ for the transport site in E1ATP. According to the model, this behavior is also expected for high concentrations of H^+^ competing with K^+^ for the site in E2P.

The possibility of K^+^ binding to E2P as the substrate, and to E1ATP as the product, explains the second order degree found for Equation 1. In the E2P conformation, the binding site exhibits high affinity for K^+^ (K_1_ in Equation 1), whereas in the E1ATP state, the cation-binding site displays low affinity for K^+^ (K_2_ in Equation 1).

In addition to cation transport, the protonation/deprotonation of amino acidic residues capable of shifting the enzyme to inactive states is contemplated (see species with nonprotonated, monoprotonated, and diprotonated states in Fig. 3*B*). For this, H^+^ ions are considered to bind under rapid equilibrium conditions, and catalytic activity is only possible through the monoprotonated forms.

The reactions in the scheme include several elementary steps. For instance, i) the step governed by *k*_*1*_ includes K^+^ binding to E2P and enzyme dephosphorylation, ii) *k*_*2*_ includes K^+^ release and E2-E1 conformational change, iii) *k*_*3*_ includes H^+^ binding and ADP dissociation, iv) the step defined by *k*_*5*_ reflects the ATPase activity detected in the absence of K^+^ and is represented by $a_{0}$ in Equation 1; this residual activity was already detected for similar P-type ATPases (17, 24). The model in Figure 3*B* can be expressed by Equation 2 (contemplating the definitions of the parameters n_i_’s and d_i_’s in terms of the k_i_’s and pK_ai_’s shown in Table S1):

(2) $$Act=\frac{n_{0}\cdot {\left[H^{+}\right]}^{3}+n_{1}\cdot {\left[H^{+}\right]}^{2}\cdot \left[K^{+}\right]}{\begin{array}{c} d_{1}\cdot \left[K^{+}\right]+d_{2}\cdot {\left[K^{+}\right]}^{2}+d_{3}\cdot {\left[H^{+}\right]}^{4}+d_{4}\cdot {\left[H^{+}\right]}^{2}\cdot {\left[K^{+}\right]}^{2}+d_{5}\cdot {\left[H^{+}\right]}^{2}{+d}_{6}\cdot {\left[H^{+}\right]}^{2}\cdot \left[K^{+}\right]+\\ {+d}_{7}\cdot \left[H^{+}\right]\cdot {\left[K^{+}\right]}^{2}{+d}_{8}\cdot \left[H^{+}\right]{+d}_{9}\cdot \left[H^{+}\right]\cdot \left[K^{+}\right]{+d}_{10}\cdot {\left[H^{+}\right]}^{3}{+d}_{11}\cdot {\left[H^{+}\right]}^{3}\cdot \left[K^{+}\right] \end{array}}$$

Note that, the lack of an independent term in the denominator of Equation 2 indicates the absence of an enzyme state simultaneously holding the transported cations, H^+^ and K^+^ (25), and validates the idea of a ping pong mechanism (where one cation is transported and released to the opposite side before the second cation binds, Fig. S1). A global fitting of the whole set of results using Equation 2 allowed to find reasonable values of *k*_i’s_ and pK_a’s_ (Table 1) and to simulate the dependence of ATPase activity. Dashed lines in Figures 1 and 2 shows that, despite its simplicity, our model provides a good description of the results, considering a stoichiometry of 1H^+^/1K^+^ per ATP hydrolyzed.

**Table 1:** Values of the rate constants and equilibrium constants (expressed as pKa) employed in the simulations

Equilibrium or rate constants	Values	Units
*k*_**1f**_	1.84E+03	min^−1^ mM^−1^
*k*_**2f**_	1.98E+04	min^−1^
*k*_**2r**_	5.70E+02	min^−1^ mM^−1^
*k*_**3f**_	6.00E+04	min^−1^ μM^−1^
*k*_**4f**_	2.87E+04	min^−1^
*k*_**4r**_	9.22E+03	min^−1^ μM^−1^
*k*_**5f**_	1.01E+03	min^−1^ μM^−1^
*pK*_**a1**_	6.68	
*pK*_**a2**_	9.00	
*pK*_**a3**_	5.22	
*pK*_**a4**_	8.77	
*pK*_**a5**_	5.22	
*pK*_**a6**_	8.77	
*pK*_**a7**_	6.68	
*pK*_**a8**_	9.00	

### Effects of K^+^ on tegoprazan inhibition of the ATPase activity

**Figure 4:**
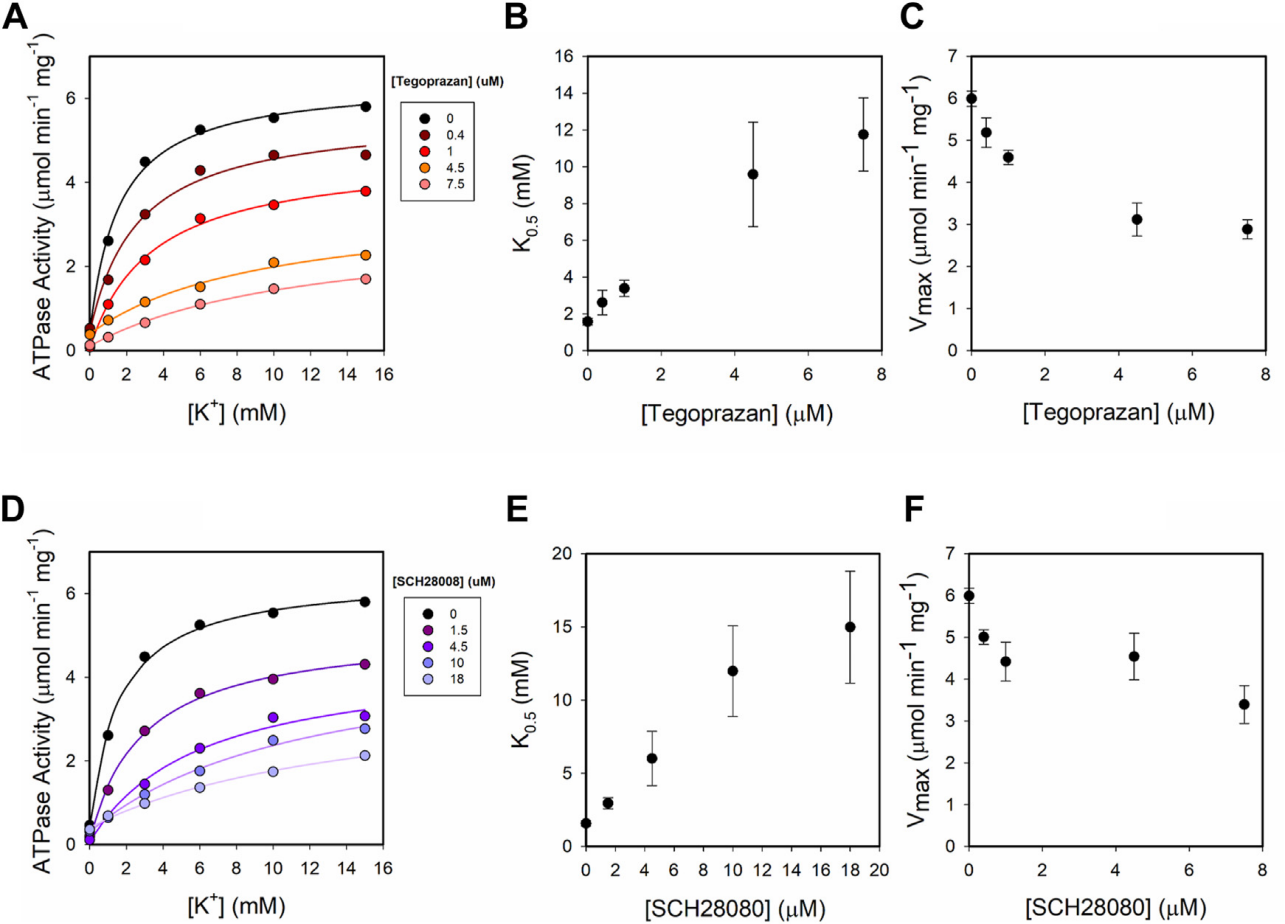
**Inhibition of H,K-ATPase activity by P-CABs.** Effect of tegoprazan (*A*) and SCH28080 (*D*) on ATPase activity for [K^+^] between 0 and 15 mM. Reaction media contained 8 μg protein ml^−1^, 0.25 mM EDTA, imidazol–Tris–HCl pH 7.2. Panels *B* and *C*, the fitted values of K_0.5_ for potassium and V_max_ for each tegoprazan concentration. *Panels E* and *F*, the fitted values of K_0.5_ for potassium and V_max_ for each SCH28080 concentration. *Continuous lines* are the plot of Equation 3. P-CAB, potassium-competitive acid blocker.

P-CABs are considered to inhibit H,K-ATPase according to a competitive mechanism. This was concluded from the evaluation of K_0.5_ and V_max_ from a Michaelis-Menten function analyzed at different [K^+^], generally constrained between 0 and 10 or 0 and 20 mM (12, 26). To test the mechanism of inhibition, we initially measured the effect of tegoprazan on the ATPase activity in a range of K^+^ concentrations of 0 to 15 mM, at a constant pH (Fig. 4*A*). The data were fit by the following function:

(3) $$Act\left(\left[K^{+}\right]\right)={\;a}_{0}+\frac{{\;V}_{\max}\;\cdot \left[K^{+}\right]}{K_{0.5}+\left[K^{+}\right]}$$

The values of the parameters of Equation 3 are shown in Figure 4, *B* and *C*. Tegoprazan induces an increase in K_0.5_ and a decrease in V_max_.

As SCH28080 is the prototype of P-CABs, we also evaluated the ATPase activity at different [SCH28080] and found similar results (Fig. 4, *D*–*F*). Here, it is important to note that our observations differ from previously published data for this type of inhibitors (21, 27, 28, 29) that reported no change in V_max_, which in turn leads to a different assignation of the inhibitory mechanism. The most likely explanation for this discrepancy is that our analysis by nonlinear regression can better determine the effect of the inhibitors on V_max_, which is difficult to detect in the Lineweaver-Burk (1/activity *versus* 1/[K^+^]) or Eadie Hofstee plots (activity *versus* activity/[K^+^]) used by other authors.

**Figure 5:**
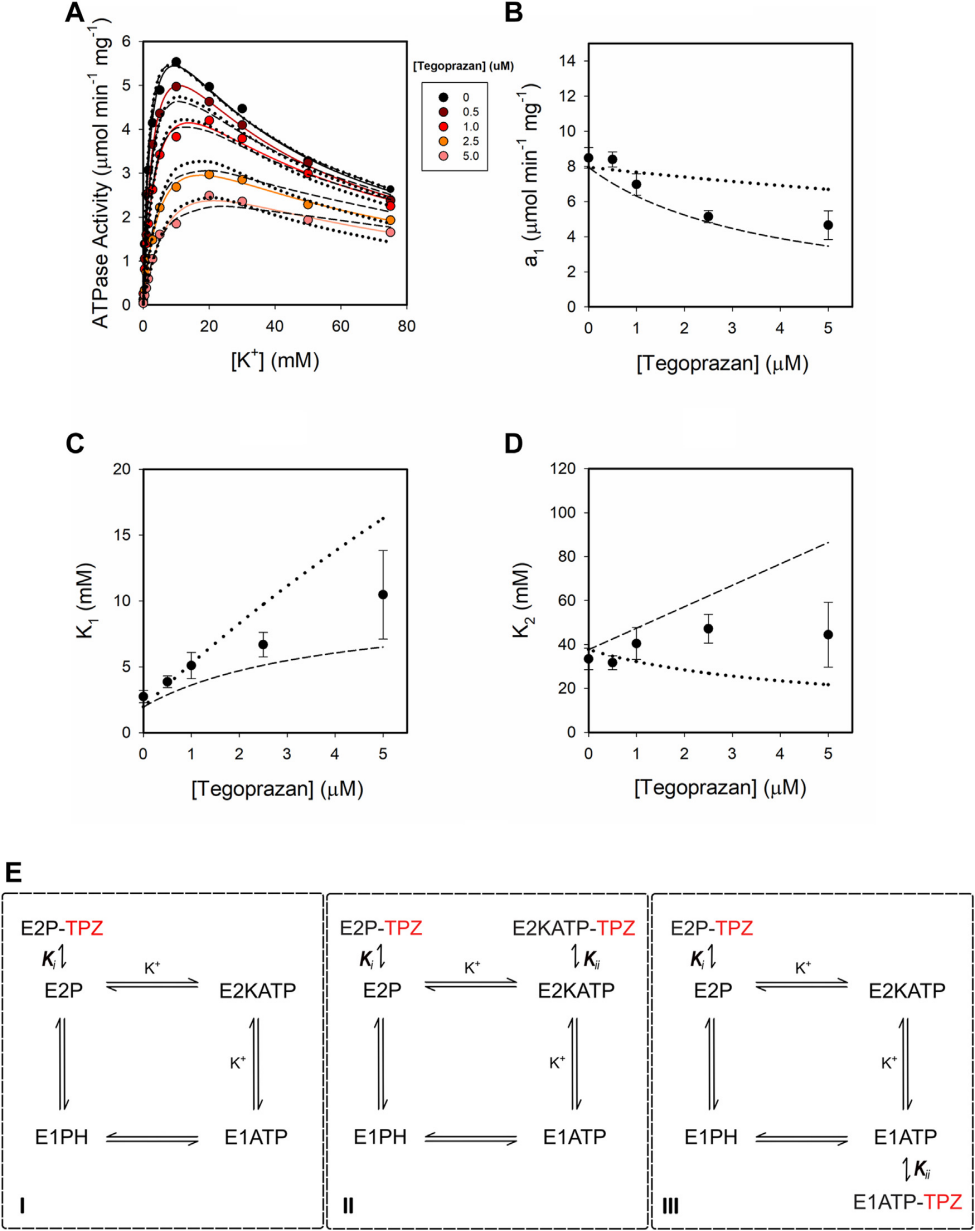
**Effect of tegoprazan on H,K-ATPase activity.***Panel A*, H,K-ATPase activity as a function of [K^+^] at different tegoprazan concentrations. *Panels B*–*D*, best fitting values of parameters from Equation 1 at each (tegoprazan). *Continuous lines* in panel A are plots of Equation 1 using the value of parameters in panels *B* to *D*. *Panel E*, three possible models to illustrate the binding of K^+^ and tegoprazan to the H,K-ATPase. Model I: tegoprazan competes with K+ for the binding to E2P. Model II tegoprazan competes with K+ for the binding to E2P and also binds to E2KATP. Model III: tegoprazan competes with K+ for the binding to E2P and also competes with K+ for the binding to E1ATP. Ki and Kii are the equilibrium constants for the binding of tegoprazan with high and low affinity, respectively. *Dotted or dashed lines* are plots of the equation from models II and III, respectively. Simulations were performed using parameters from Table 1 and K_i_ (μM) = 0.41 and K_ii_ (μM) = 3.55 for model II or 0.56 μM, and 2.70 μM for model III.

Yet, given the dual effect of K^+^ that we have shown in the first part of this work (Fig. 1*A*), it seems prudent to test a wider range of [K^+^] to investigate the mechanism of enzyme–tegoprazan interaction. Figure 5*A*, shows the ATPase activity against [K^+^] between 0 and 75 mM. The second-order rational function of [K^+^] (Equation 1) was fitted for each tegoprazan condition and the best-fitting values of the parameters, $a_{1}$, K_1_, and K_2_ are displayed, as a function of [tegoprazan], in Figure 5, *B*–*D*. It can be seen an increase in K_1_ with tegoprazan, which, according to the H,K-ATPase model (Fig. 3), is probably related to the binding of tegoprazan to E2P. The theoretical maximal activity ($a_{1}$) decreases as a function of the inhibitor concentration, and K_2_ values show a slight increase in this concentration range of K^+^.

On the basis of our observations and the competition stated between the K^+^ and P-CABs (30), we propose in Figure 5*E* three different models for the analysis of the interaction between tegoprazan, K^+^, and H,K-ATPase. One model considers the binding of tegoprazan only to E2P (model I). Model II considers the binding of tegoprazan to E2P and to E2KATP, and Model III declares that tegoprazan can bind to E2P and to E1ATP. The corresponding equations for the ATPase activity are summarized in Table 2. It is possible to reorganize the equations to express $a_{1}$ as a function of [tegoprazan] for each model. Table 2 shows that in Model I, $a_{1}$ is independent of the inhibitor concentration, excluding this model from further considerations. Accordingly, to choose the best model, we fitted the equations derived from models II and III and calculated the corrected asymptotic information criterion (AICc) values. Although the values obtained were similar (dotted *versus* dashed lines in Fig. 5*A*), the lower AICc was found for model III, which predicts that tegoprazan can bind to E2P and, with lower affinity, to E1ATP.

**Table 2:** Equations for the initial velocity from models I, II, and III

Model	$v_{i}$	$a_{1}$
I	$\frac{n_{0}+n_{1}\cdot \left[K^{+}\right]}{d_{0}+d_{1}\cdot \left[Inh\right]+d_{2}\cdot \left[K^{+}\right]+d_{3}{\cdot \left[K^{+}\right]}^{2}}$	$\frac{n_{1}}{d_{2}}$
II	$\frac{n_{0}+n_{1}\cdot \left[K^{+}\right]}{d_{0}+d_{1}\cdot \left[Inh\right]+d_{2}\cdot \left[Inh\right]\cdot \left[K^{+}\right]+d_{3}{\cdot \left[K^{+}\right]+d_{4}\cdot \left[Inh\right]\cdot \left[K^{+}\right]}^{2}+d_{5}\cdot {\left[K^{+}\right]}^{2}}$	$\frac{n_{1}}{d_{2}\cdot \left[Inh\right]+d_{3}}$
III	$\frac{n_{0}+n_{1}\cdot \left[K^{+}\right]}{d_{0}+d_{1}\cdot \left[Inh\right]+d_{2}\cdot \left[Inh\right]\cdot \left[K^{+}\right]+d_{3}{\cdot \left[K^{+}\right]+d_{4}\cdot \left[K^{+}\right]}^{2}}$	$\frac{\;n_{1}}{\;d_{2}\cdot \left[Inh\right]+d_{3}}$

The terms can be reorganized and a_1_ from Equation 1 can be expressed for each model. The meaning of n_i's_ and d_i's_ are detailed in Table S2.

Since the scheme in Figure 3 is a simplification of the catalytic cycle, we should not rule out the possibility that an intermediate state between E2KATP and E1ATP is the one that binds tegoprazan with low affinity. Fitting model III, we can estimate the apparent affinity constant for tegoprazan and the species E2P and E1ATP: (μM) 0.56 ± 0.04 and 2.70 ± 0.24, respectively, at pH 7.2.

### Effect of H^+^ on tegoprazan inhibition

**Figure 6:**
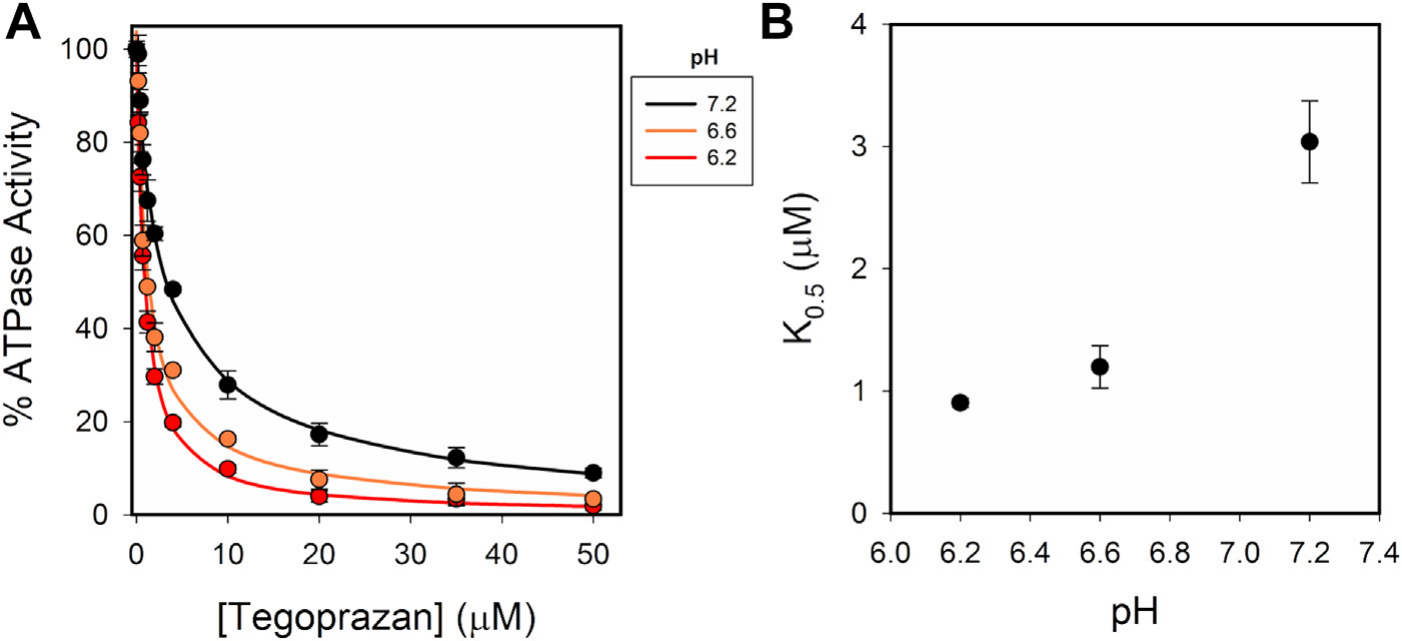
**Effect of pH on the inhibition of H,K-ATPase by tegoprazan.** Reaction media contained 8 μg protein ml^−1^, 0.25 mM EDTA, and 10 mM KCl. Panel *B* shows the K_0.5_ calculated from curves in panel *A*. *Continuous lines* were drawn according to $\%\;\text{ATPase}\;\text{Act}=\frac{a_{1}\cdot K_{1}}{\left[\text{Tegoprazan}\right]+K_{1}}+\frac{a_{2}\cdot K_{2}}{\left[\text{Tegoprazan}\right]+K_{2}}$.

Here, we measured ATPase activity as a function of tegoprazan concentrations at a fixed [K^+^] by varying the [H^+^] (Fig. 6). As already published for other P-CABs (26, 31), we observed that H^+^ increases the apparent affinity for tegoprazan; the K_0.5_ decreases from (μM) 3.25 ± 0.29 to 0.89 ± 0.04 between pH 7.2 and pH 6.2. Given that, tegoprazan is a weak base that shifts to the protonated state with H^+^, and that the H,K-ATPase has several ionizable groups, it seems difficult to elucidate the level at which H^+^ exerts its effect (on the protein or on the inhibitor molecule) on the sole basis of the present results. A way to elucidate this issue is through a structural analysis using molecular dynamics (MD).

### Molecular dynamics

**Figure 7:**
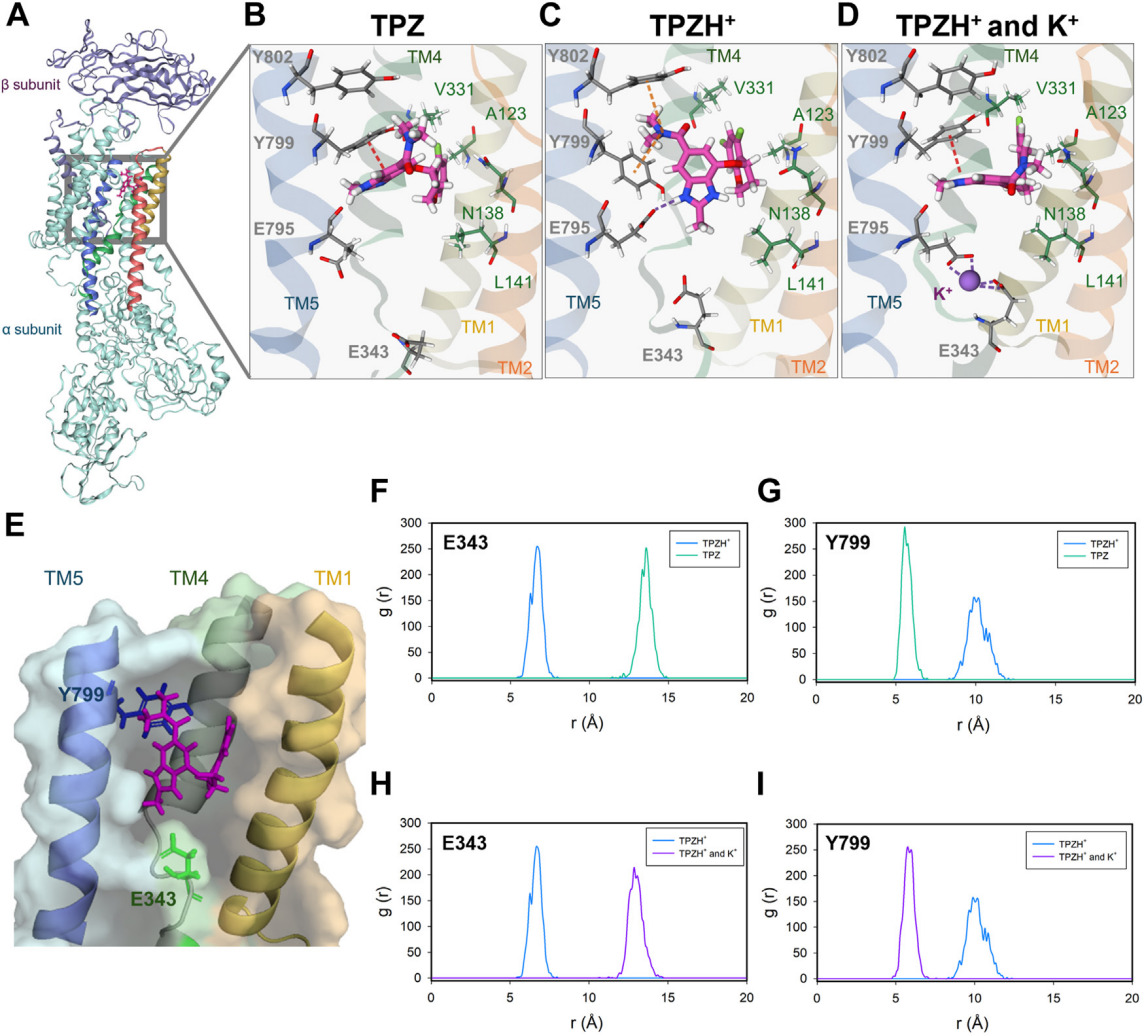
**Effect of H**^**+**^**and K**^**+**^**on tegoprazan binding.** The model was constructed from the E2 conformation PDB 7W47 (panel *A*). Representative poses of protonated tepograzan (panel *B*), unprotonated tepograzan (panel *C*), and protonated tegoprazan with K^+^ at the binding site (*D*) are illustrated. Transmembrane (TM) helices and residues are represented, respectively, as *ribbons* and *sticks*. Interactions are indicated as *dotted lines*: ionic (*purple*), cation-π (*orange*), and π-π stacking (*red*). Residues shown in *green* are involved in interactions with tegoprazan, which remain almost unchanged under different conditions. Panel E shows the surface of the luminal pocket of H,K-ATPase and tegoprazan (*pink sticks*). The position of Y799 at the entrance and E343 at the *bottom* of the binding pocket are highlighted as *blue* and *green sticks*, respectively. Panels F to I present the calculated RDFs between the geometric center 1, defined as the carbon of the methyl group in the benzoimidazole ring of tegoprazan, and center 2, defined as the alpha carbon of Y799 or the alpha carbon of E343. RDF, radial distribution function.

Seeking an explanation for the enzyme inhibition at different pH, the structures obtained with unprotonated (TPZ) and protonated tegoprazan (TPZH^+^), under conditions where the K^+^ binding site is empty, were subjected to MD simulations for 100 ns (Fig. S2). When we measured the θ dihedral angle for the states obtained from the last 40 ns of the MD simulations, we observed that the protonated molecule presents a lower value than the neutral form: 40 to 75° (TPZH^+^) *versus* 140 to 160° (TPZ). The structures show that while the hydrophobic contacts between the difluorochromanyl ring and the residues in TM1, TM2, and TM4 are fairly similar for the protonated and nonprotonated tegoprazan, there is a significant difference in the pose of the benzoimidazole ring among both states of the inhibitor (see panels B *versus* C in Fig. 7). The benzoimidazole ring of protonated tegoprazan shows a rotation that probably helps it to fit deeper into the binding pocket. To determine the relative position of tegoprazan within the protein pocket, the radial distribution function (RDF) was estimated. We have used the locations of Y799 and E343 as reference points, since these residues are located at the entrance to and deep within the pocket, respectively (Fig. 7*E*). RDFs were measured between the alpha carbons of these residues and the carbon of the methyl group in the benzoimidazole ring of tegoprazan. As shown in Figure 7, *F* and *G*, when tegoprazan is protonated, it is closer to E343 than the unprotonated molecule (∼6.6 Å *versus* ∼14 Å). Conversely, the nonprotonated form is close to Y799 (∼5.5 Å *versus* ∼10 Å). These observations are consistent with the lower K_0.5_ obtained at lower pH in Figure 6*B*.

Since P-CABs have been proposed to bind within the cavity leading to, or substantially into, the K^+^ binding site (29), we inspected the impact of bound K^+^ during the simulations. Figure 7, *C versus D*, shows important differences in tegoprazan position. Concerning E795, whereas this residue binds K^+^ through the carbonyl oxygen of the side chain; in the absence of K^+^, the protonated benzimidazole ring of tegoprazan is able to interact by a salt bridge with the carbonyl oxygen of the E795 side chain, that is, tegoprazan can go deeper into the cavity. In the absence of K^+^ a cation-π interaction appears between Y802 and the amide group of the protonated tegoprazan. The data also show that the interaction between tegoprazan and Y799 changes by a π stacking (with K^+^) for a cation- π (without K^+^) one. Inspection of RDFs in Figure 7, *H* and *I*, indicates that bound K^+^ causes inhibitor displacement toward the luminal cavity entrance. These features can reasonably explain the decrease in the affinity for K^+^ with tegoprazan concentration shown in Figure 5*C*.

## Discussion

To fully explore the inhibition of H,K-ATPase by tegoprazan, in this work, we first describe the enzyme kinetics in a noncompartmentalized system, where K^+^ and H^+^ can activate but also inhibit ATPase activity.

Although there has been a lot of controversy about the stoichiometry during the catalytic cycle, the analysis of steady-state results allows us to explain the non-Michaelis-Menten kinetics of H,K-ATPase through a ping-pong mechanism that considers a stoichiometry of 1 H^+^, 1 K^+^ per hydrolyzed ATP. Regarding the various theories concerning H^+^ transport through membrane proteins (18, 19, 20), our model explains a "pump mechanism" for cation transport, where K^+^ and H^+^ are transported by an alternating access mechanism between inward-facing E1 and outward-facing E2 conformations.

The proposed scheme is compatible with previous observations: i) the K^+^-induced dephosphorylation observed by Wallmark *et al.* (21) and ii) the increased rate and extent of phosphorylation induced at lower pH and the fact that 5 mM K^+^ changes the rate of phosphorylation under alkaline but not under acidic conditions (32). In the model, the effects of H^+^ as the transported cation and affecting the ionization states of the protein are considered. Importantly, the peak shift showed in the pH profile may be useful in assessing whether H^+^ is the counter-transported cation, as proposed for certain members of the P-type ATPase group (22).

A detailed analysis of the inhibition kinetics denotes the binding of tegoprazan to at least two different intermediate states during the catalytic cycle. This highlights the need for caution when considering inhibition by P-CABs to avoid wrongly assuming a classical K^+^-competitive mechanism (33). In this sense, the decrease in “V_max,_” which was previously observed for certain ligands on enzymes with point mutations, was interpreted as a change in the inhibition mechanism (34), while that observation, as is evident from this work, can be explained simply as a change in the affinity between the H,K-ATPase and P-CAB molecules.

The question then arises, which intermediates can bind tegoprazan? A careful evaluation of physically possible models was carried out to find the intermediates capable of binding to tegoprazan that led to the formation of the dead-end complexes. The lower AICc value is found for model III, where tegoprazan can bind to E2P and to E1ATP. Nevertheless, we cannot ignore the crystal structure where the protein is simultaneously bound to K^+^ and tegoprazan (35), which could have no functional consequences due to the particular conditions under which the crystals were obtained.

To find a structural explanation for the enzyme activity results, we carried out MD simulations and analyze the effect of H^+^ and K^+^ on the interaction between H,K-ATPase and tegoprazan.

Regarding the effect of H^+^, our results from MD simulations show that protonation of tegoprazan induces a change in the pose of the inhibitor, particularly of the benzimidazole ring, at the binding cavity. This allows interacting with a deeper zone within the transmembrane helices, as suggested by the RDF values. Since this is a reasonable explanation for the higher apparent affinity we have found at low pH, we cannot rule out the possibility that protonation of some residues could somehow alter the conformation of the enzyme.

In the design of novel benzimidazole derivatives by structure-based optimization methods, no attention has been paid to the comparison of conformations in which K^+^ is bound with those in which the cation site is free (13, 33). Indeed, although it has been proposed that tegoprazan binds superficially, this is based on the observation of structures containing simultaneously tegoprazan and K^+^. Here, we show that K^+^ induces an important change in the position of tegoprazan, which in the absence of K^+^ moves further inward. According to thermodynamic principles, this is consistent with the increase in the apparent dissociation constant K_1_ for K^+^ with (tegoprazan) that we found in kinetic experiments.

In general, this work highlights the importance of an exhaustive knowledge of the effect of transported cations on the H,K-ATPase for the design of new inhibitors.

## Experimental procedures

### Enzyme preparation

We prepared H,K-ATPase (EC 7.2.2.19)-enriched membrane vesicles according to Sachs *et al.* (36), with slight modifications. The number of nucleotide sites measured under optimal conditions (2.2 mM ATP, 4 mM MgCl_2_, 0.25 mM EDTA in 25 mM imidazole–HCl, pH 7.4 at 25 °C) was 1.4 nmol per mg of protein. This preparation is essentially free of contamination with Na,K-ATPase, as evidenced by the lack of ouabain-sensitive activity. 0.4 to 0.6 mg alamethicin per milligram protein was used to permeabilize the vesicles.

### Reagents

Alamethicin, choline chloride, Na_2_VO_4_, ATP, and SCH28080 were from Sigma. Tegoprazan was a kind gift from HK inno.N Corporation. SCH28080 and tegoprazan were dissolved in dimethyl sulfoxide (DMSO).

### ATPase activity

ATP hydrolysis by H,K-ATPase was measured by following the time course of Pi release at 37 °C; linearity was always checked in order to ensure initial rate conditions. Pi concentration was determined according to the method described by Baginski *et al.* (37) Incubations were performed at 37 °C in media containing 8 μg protein ml^−1^, 2 mM ATP, 4 mM MgCl_2_, and 0.25 mM EDTA in 20 mM imidazole – 20 mM Tris (varying pH) in a final volume of 0.25 ml. KCl concentrations varied between 0 and 100 mM, using choline chloride to adjust total monovalent salt concentration to 150 mM. Reaction blanks were determined by measuring ATP hydrolysis in media containing vanadate. For experiments at variable pH, a buffer combination of 20 mM imidazole-HCl and 20 mM Tris–HCl was used. Tegoprazan and SCH28080 were dissolved in DMSO. The final DMSO concentration in the reaction media was 1%.

### Data analysis and kinetic models

Empirical equations of the form

(4) $$Act\left(\left[K^{+}\right]\right)=\frac{a_{0}+a_{1}\cdot \frac{\left[K^{+}\right]}{K_{1}}+a_{2}\cdot \frac{{\left[K^{+}\right]}^{2}}{K_{1}K_{2}}+\ldots +a_{n}\cdot \frac{{\left[K^{+}\right]}^{n}}{{K_{1}K_{2}K}_{n}}}{1+\frac{\left[K^{+}\right]}{K_{1}}+\frac{{\left[K^{+}\right]}^{2}}{K_{1}\cdot K_{2}}+\ldots +a_{d}\cdot \frac{{\left[K^{+}\right]}^{d}}{{K_{1}K_{2}K}_{d}}}$$

were fitted to the data of ATPase activity by nonlinear regression based on the Gauss–Newton algorithm by commercial programs (38). *K*_*is*_ parameters are apparent dissociation constants expressed in concentration units, whereas the $a_{is}$ parameters are coefficients with units of activity. A methodical exploration of the equation that best fits each set of results was performed. We first determined the degree of the polynomials in the numerator and the denominator (considering the restriction of *n* ≤ *d* and *m* ≤ *d* to conform to steady-state equations for enzymes) and then evaluated the contribution of terms of intermediate degree. The goodness of fit of a given equation to the experimental results was evaluated by the AICc (39) defined as

(5) $${AIC}_{c}=N\cdot \;\ln\left(\frac{SS}{N}\right)+\frac{2\cdot \left(K+1\right)\cdot N}{\left(N-K-2\right)}$$

with *N*: number of data, *K*: number of parameters, and *SS*: sum of weighted square residual errors. This criterion allows one to select among multiple equations, the one that best describes the behavior of the experimental data using the least number of parameters, that is the lower value of AICc.

The equations of activity as a function of ligands for each kinetic model were attained using the Mathematica for Windows program. To determine the parameters that will be used to simulate the data, the rate constants and pKa values were fitted to the results using a procedure based on the Gauss–Newton algorithm until the SD of the residual errors reaches a minimal value.

### Computational analysis

#### Preparation of tegoprazan molecule

##### Protonation site of tegoprazan

**Figure 8:**
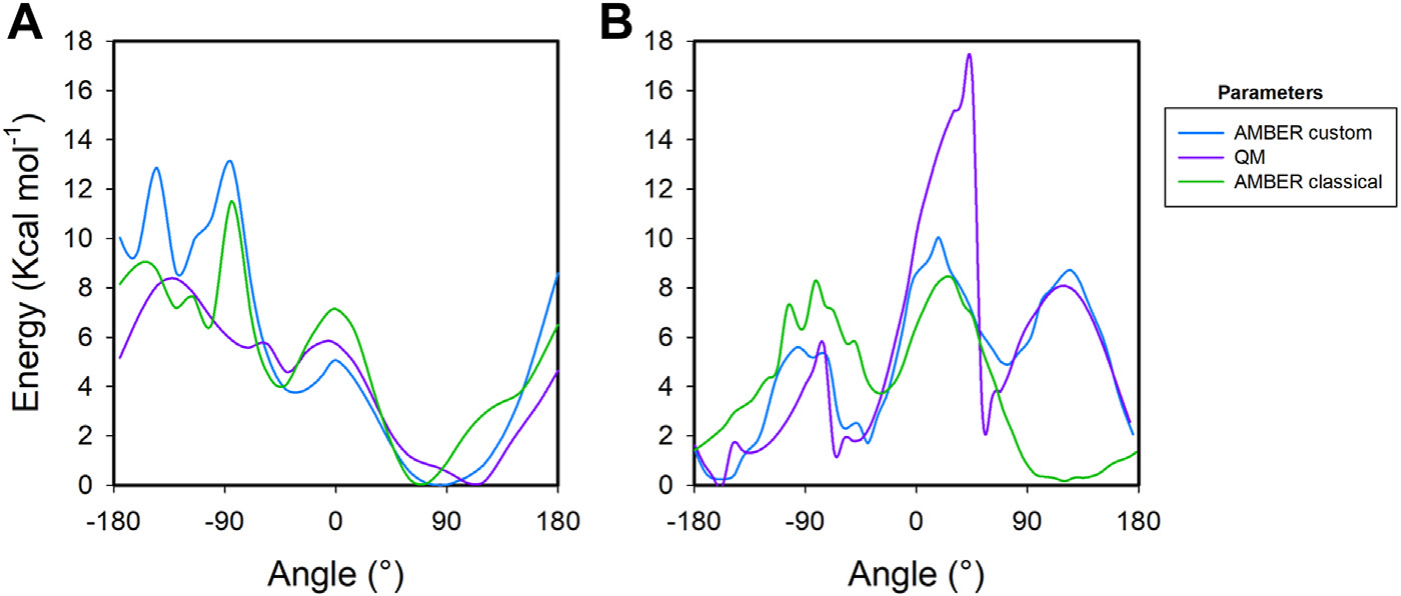
**Tegoprazan molecule parameterization.***Panel A*, structure of tegoprazan. *Purple arrows* indicate the two putative sites of protonation; *green arrow* shows the θ dihedral angle. *Panel B*, Energy profile as a function of the θ dihedral angle values for unprotonated (*A*) and protonated (*B*) tegoprazan.

Parameters for MD simulations were derived from geometry and charge optimizations performed with Gaussian09 (40) and antechamber from AMBERTOOLS package (41). B3LYP functional at 6-31G∗ basis was used for geometry optimizations and charges were calculated with HF at 6-31G∗. Quantum mechanics (QM) calculations were provided to antechamber to calculate charges *via* restrained electrostatic potential. To assess the protonation site of tegoprazan, proton affinities (PAs) were computed for two putative protonation sites: the imidazole ring and the amide nitrogen (Fig. 8). PA is defined as the negative of the enthalpy change, ΔH, for the gas phase reaction between a proton and a chemical species. Calculated PA values were 242.112 kcal/mol and 217.433 kcal/mol, for the imidazole ring and the N atom of amide group, respectively. This difference indicates that protonation is 1.5 × 10^18^ more likely to occur in the imidazole ring than in the amide group.

##### Optimizations of the *θ* dihedral angle

First, a dihedral angle scan was carried out in Gaussian09 to obtain a reference QM energy profile. After that, the dihedral angle scan was also performed with *gaff2* AMBER force field (Amber default). Finally, AMBER dihedral parameters were optimized then with a *python* genetic algorithm to fit the classical energy profile to the QM energy profile (Amber custom), in order to render similar minima and kinetic barriers (http://www.ub.edu/bl/2017/03/16/small-molecule-dihedrals-parametrization/). Figure 8, *B* and *C*, show that while Amber default parameters can reproduce the QM energy profile for unprotonated tegoprazan, they cannot provide a proper description for the protonated molecule. For this reason, custom parameters were developed for a quantum-like description of θ in a classical force field. After this procedure, we obtained an energy profile like the QM energy profile for the protonated tegoprazan regarding wells and peak positions (see orange and blue lines in Fig. 8*C*).

The new force field parameters obtained in this work are available at https://github.com/GeraZerbetto/tegoprazan_dih_params.

#### MD simulations

The H,K-ATPase model was built from the E2 conformation of H,K-ATPase (PDB 7W47). Crystallographic water molecules and Mg^2+^ ions were removed. Rb^+^ ions were replaced by K^+^, and beryllium and fluorine atoms were removed. The protein was embedded in a fully hydrated 135Å × 135Å 1-palmitoyl-2-oleoyl-sn-glycero-3-phosphocholine bilayer using the membrane builder tool provided in the CHARMM-GUI website (42, 43, 44). Approximately, 183000 TIP3P explicit water molecules and about 520 sodium and chloride ions were added to achieve a concentration of 150 mM. Total box dimensions in all simulations were approximately 135Å × 135Å × 330Å (X,Y,Z). A minimization stage was performed prior to the heating stages. The system was first heated from 0 to 100 K with all the lipids under weak harmonic constraints and then heated from 100 to 303 K with an anisotropic Berendsen weak coupling barostat to equilibrate the pressure under the same harmonic constraints. Thereafter, constrain-free MD was run in an NPT ensemble with full periodic boundary conditions. Nonbonded interactions (electrostatic and Lennard-Jones) were simulated with a cut-off of 10Å. Electrostatic interactions were simulated using Particle Mesh Ewald for long-range interactions. All simulations were performed using AMBER18 MD simulations package and parameters from AMBER14SB, LIPID17, and GLYCAM06j−1 force fields (45). Covalent hydrogen bonds were constrained using SHAKE algorithm, allowing a time step of 2 fs. The simulations were performed at a temperature of 303 K using a Langevin thermostat and pressure was kept at 1 bar by an anisotropic Berendsen barostat. Equilibrium simulations were carried out during 100 ns. Every model render was obtained with the program VMD. Three independent replicas were run for each system starting from different initial velocities.

## Data availability

Most of the data described are contained within the article. Any additional data or further description will be shared upon request to the corresponding author.

All authors have given approval to the final version of the article.

## Supporting information

This article contains supporting information (24).

## Conflict of interest

The authors declare that they have no conflicts of interest with the contents of this article.
