# Supplementary material for: How ligands modulate the gastric H,K-ATPase activity and its inhibition by tegoprazan
Source: J Biol Chem. 2024 Nov 14;300(12):107986. doi: 10.1016/j.jbc.2024.107986 (PMC11697777; doi:10.1016/j.jbc.2024.107986)
Supplement: Supporting information [file mmc1.docx]

**HOW LIGANDS MODULATE the gastric H,K-ATPASE ACTIVITY AND ITS INHIBITION BY TEGOPRAZAN**

N.T. Cerf, G. Zerbetto de Palma, N.U. Fedosova, C. V. Filomatori, R. C. Rossi, S.E. Faraj, M.R. Montes.

**List of the material included**

Figure S1: A scheme for a ping pong mechanism.

Figure S2: Root mean square deviation (RMSD) for tegoprazan and H,K-‑ATPase interaction.

Table S1: Meaning of *n_i_* and *d_i_* coefficients of Equation 4 (main text) in terms of the model of Figure 4.

Tables S2, A, B, and C: Meaning of *n_i_* and *d_i_* coefficients for Models I, II and III of Figure 5.

**Figure S1**


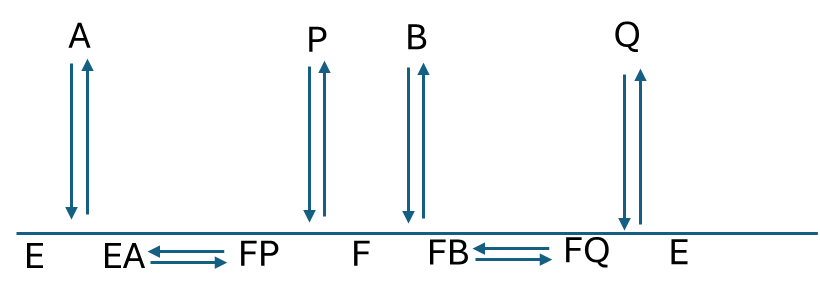


Figure S1. A scheme for a ping pong mechanism

In a ping pong mechanism (Figure S1), the product of the first reaction step is released from the enzyme before the other substrate binds.

In the scheme substrate A is equivalent to the extracellular K^+^, first product P is equivalent to intracellular K^+^, substrate B is equivalent to intracellular H^+^, and, second product Q is equivalent to extracellular H^+^.

For this model, the increase in K_M_ as a function of one substrate is expected to be proportional to the increase in the maximal activity: the ratio K_M_/V_max_ is constant (24).

**Figure S2**


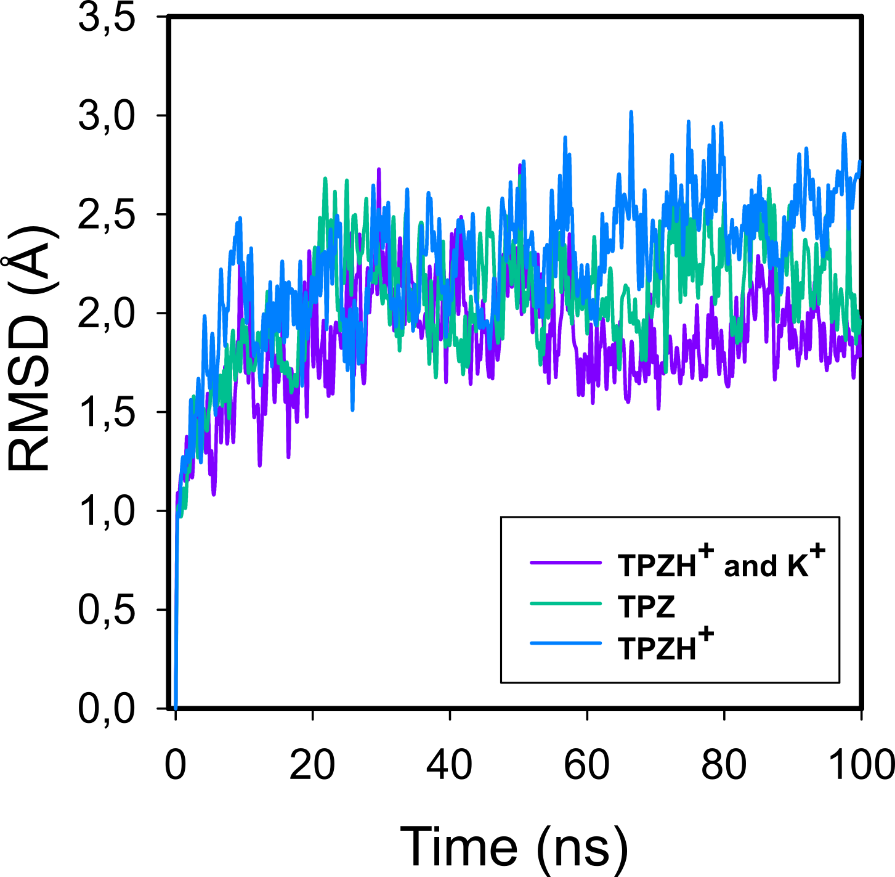


Figure S2 Root mean square deviation (RMSD) for tegoprazan and H,K-ATPase interaction. RMSD were calculated for 100 ns for protonated Tegoprazan in the absence (TPZH^+^) or presence (TPZH^+^ and K^+^) of K^+^ and unprotonated Tegoprazan in the absence of K^+^.

**Table S1**

| Parameter | Definition |
| --- | --- |
| $n_{0}$ | $\left[ E_{t} \right]\cdot k_{2f}\cdot k_{3f}\cdot k_{4f}\cdot k_{5f}\cdot{Ka}_{2}\cdot{Ka}_{4}\cdot{Ka}_{6}\cdot{Ka}_{8}$ |
| $n_{1}$ | $\left[ E_{t} \right]\cdot k_{1f}\cdot k_{2f}\cdot k_{3f}\cdot k_{4f}\cdot{Ka}_{2}\cdot{Ka}_{4}\cdot{Ka}_{6}\cdot{Ka}_{8}$ |
| $d_{1}$ | $k_{1f}\cdot k_{2r}\cdot k_{4f}\cdot{Ka}_{1}\cdot{Ka}_{2}\cdot{Ka}_{4}\cdot{Ka}_{6}\cdot{Ka}_{8}$ |
| $d_{2}$ | $k_{1f}\cdot k_{2f}\cdot k_{4f}\cdot{Ka}_{2}\cdot{Ka}_{3}\cdot{Ka}_{4}\cdot{Ka}_{6}\cdot{Ka}_{8}$ |
| $d_{3}$ | $k_{2f}\cdot k_{3f}\cdot{(k}_{4r}+k_{5f})\cdot{Ka}_{2}{\cdot Ka}_{4}\cdot{Ka}_{8}$ |
| $d_{4}$ | $k_{1f}\cdot k_{2r}\cdot k_{4f}\cdot{Ka}_{4}\cdot{Ka}_{6}\cdot{Ka}_{8}$ |
| $d_{5}$ | $k_{2f}\cdot{Ka}_{2}{\cdot Ka}_{4}\cdot(k_{4f}\cdot\left( k_{3f}+k_{5f} \right)+k_{3f}\cdot\left( k_{4r}+k_{5f} \right)\cdot{Ka}_{5}){\cdot Ka}_{6}\cdot{Ka}_{8}$ |
| $d_{6}$ | ${Ka}_{2}\cdot(k_{1f}\cdot k_{2f}\cdot k_{4f}+k_{1f}\cdot k_{3f}\cdot(k_{2f}+k_{4f})\cdot{Ka}_{4}+k_{2r}\cdot k_{4f}\cdot k_{5f}{\cdot Ka}_{4}){\cdot Ka}_{6}\cdot{Ka}_{8}$ |
| $d_{7}$ | $k_{1f}\cdot k_{2r}\cdot k_{4f}\cdot{Ka}_{2}\cdot{Ka}_{4}\cdot{Ka}_{6}\cdot{Ka}_{8}$ |
| $d_{8}$ | $k_{2f}\cdot k_{4f}\cdot{Ka}_{2}\cdot{Ka}_{4}\cdot{Ka}_{6}\cdot(k_{5f}\cdot{Ka}_{3}+k_{3f}\cdot{Ka}_{7})\cdot{Ka}_{8}$ |
| $d_{9}$ | ${Ka}_{2}\cdot{Ka}_{4}\cdot( {k_{1f}{\cdot k}_{3f}\cdot k_{4f}\cdot Ka}_{1}+ k_{2r}\cdot k_{4f}\cdot k_{5f}\cdot{Ka}_{1}+k_{1f}{\cdot k}_{2f}\cdot\left( k_{4f}+k_{3f}\cdot{Ka}_{5} \right))\cdot{Ka}_{6}\cdot{Ka}_{8}$ |
| $d_{10}$ | $k_{2f}\cdot k_{4f}\cdot k_{5f}\cdot{Ka}_{2}{\cdot Ka}_{6}\cdot{Ka}_{8}+k_{2f}\cdot k_{3f}\cdot{Ka}_{2}{\cdot Ka}_{4}{\cdot Ka}_{6}\cdot(k_{4f}+(k_{4r}+k_{5f})\cdot{Ka}_{8})$ |
| $d_{11}$ | $k_{1f}\cdot k_{2f}\cdot k_{3f}\cdot{Ka}_{2}\cdot{Ka}_{4}\cdot{Ka}_{8}+k_{4f}\cdot(k_{1f}\cdot k_{3f}+k_{2r}{\cdot k_{5f})\cdot Ka}_{4}\cdot{Ka}_{6}\cdot{Ka}_{8}$ |

Table S1: Meaning of *n_i_* and *d_i_* coefficients of Equation 4 (main text) in terms of the model of Figure 3.

**Table S2A**

| Parameter | Definition |
| --- | --- |
| $n_{0}$ | $\left[ E_{t} \right]\cdot\left[ H \right]^{3}\cdot k_{2f}\cdot k_{3f}\cdot k_{4f}\cdot k_{5f}\cdot{Ka}_{2}\cdot{Ka}_{4}\cdot{Ka}_{6}\cdot{Ka}_{8}\cdot K_{i}$ |
| $n_{1}$ | $\left[ E_{t} \right]\cdot\left[ H \right]^{2}\cdot k_{1f}\cdot k_{2f}\cdot k_{3f}\cdot k_{4f}\cdot{Ka}_{2}\cdot{Ka}_{4}\cdot{Ka}_{6}\cdot{Ka}_{8}\cdot K_{i}$ |
| $d_{0}$ | $\left[ H \right]\cdot k_{2f}\cdot{Ka}_{2}\cdot(\left[ H \right]^{3}\cdot k_{3f}\cdot(k_{4r}+{k_{5f})\cdot Ka}_{4}\cdot{Ka}_{8}+\left[ H \right]\cdot{Ka}_{4}\cdot(k_{4f}\cdot$  $(k_{3f}+k_{5f})+k_{3f}(k_{4r}+k_{5f})\cdot{Ka}_{5})\cdot{Ka}_{6}\cdot{Ka}_{8}+k_{4f}{\cdot Ka}_{4}\cdot{Ka}_{6}\cdot(k_{5f}\cdot{Ka}_{3}+$  $k_{3f}\cdot{Ka}_{7})\cdot{Ka}_{8}+\left[ H \right]^{2}\cdot{Ka}_{6}\cdot(k_{4f}\cdot k_{5f}\cdot{Ka}_{8}+k_{3f}\cdot{Ka}_{4}\cdot(k_{4f}+(k_{4r}+k_{5f})\cdot{Ka}_{8})))\cdot K_{i}$ |
| $d_{1}$ | $\left[ H \right]^{2}\cdot k_{2f}\cdot k_{3f}\cdot k_{4f}\cdot{Ka}_{2}\cdot{Ka}_{4}\cdot{Ka}_{6}\cdot{Ka}_{8}$ |
| $d_{2}$ | $k_{1f}\cdot k_{2f}\cdot k_{4f}\cdot{Ka}_{2}\cdot{Ka}_{3}\cdot{Ka}_{4}\cdot{Ka}_{6}+\left[ H \right]^{2}\cdot{Ka}_{2}\cdot(k_{1f}\cdot k_{2f}\cdot k_{4f}+k_{1f}\cdot k_{3f}\cdot(k_{2f}+$  $k_{4f})\cdot{Ka}_{4}+k_{2r}\cdot k_{4f}\cdot k_{5f}\cdot{Ka}_{4})\cdot{Ka}_{6}+\left[ H \right]\cdot{Ka}_{2}\cdot{Ka}_{4}\cdot(k_{2r}\cdot k_{4f}\cdot k_{5f}\cdot{Ka}_{1}+$  $k_{1f}\cdot(k_{2f}\cdot k_{4f}+k_{3f}\cdot k_{4f}\cdot{Ka}_{1}+k_{2f}\cdot k_{3f}\cdot{Ka}_{5})) \cdot{Ka}_{6}+\left[ H \right]^{3}\cdot{Ka}_{4}(k_{2r}\cdot k_{4f}\cdot k_{5f}\cdot{Ka}_{6}+$  $k_{1f}\cdot k_{3f}(k_{2f}\cdot{Ka}_{2}+k_{4f}\cdot{Ka}_{6})) {Ka}_{8}\cdot K_{i}$ |
| $d_{3}$ | $k_{1f}\cdot k_{2r}\cdot k_{4f}\cdot(\left[ H \right]^{2}+(\left[ H \right]+{Ka}_{1})\cdot{Ka}_{2})\cdot{Ka}_{4}\cdot{Ka}_{6}\cdot{Ka}_{8}\cdot K_{i}$ |

Table S2A: Meaning of *n_i_* and *d_i_* coefficients for Models I in Figure 5. Where the Equation for Model I is:

$$\frac{n_{0}+ n_{1}[K]}{d_{0}+ d_{1}\left[ Inh \right]+ d_{2}\left[ K \right]+ d_{3}{[K]}^{2}}$$

**Table S2B**

| Parameter | Definition |
| --- | --- |
| $n_{0}$ | $\left[ E_{t} \right]\cdot\left[ H \right]^{3}\cdot k_{2f}\cdot k_{3f}\cdot k_{4f}\cdot k_{5f}\cdot{Ka}_{2}\cdot{Ka}_{4}\cdot{Ka}_{6}\cdot{Ka}_{8}\cdot K_{i}\cdot K_{ii}$ |
| $n_{1}$ | $\left[ E_{t} \right]\cdot\left[ H \right]^{2}\cdot k_{1f}\cdot k_{2f}\cdot k_{3f}\cdot k_{4f}\cdot{Ka}_{2}\cdot{Ka}_{4}\cdot{Ka}_{6}\cdot{Ka}_{8}\cdot K_{i}\cdot K_{ii}$ |
| $d_{0}$ | $\left[ H \right]\cdot k_{2f}\cdot{Ka}_{2}\cdot(\left[ H \right]^{3}\cdot k_{3f}\cdot(k_{4r}+{k_{5f})\cdot Ka}_{4}\cdot{Ka}_{8}+\left[ H \right]\cdot{Ka}_{4}\cdot(k_{4f}\cdot(k_{3f}+k_{5f})+$  $k_{3f}(k_{4r}+k_{5f})\cdot{Ka}_{5})\cdot{Ka}_{6}\cdot{Ka}_{8}+k_{4f}{\cdot Ka}_{4}\cdot{Ka}_{6}\cdot(k_{5f}\cdot{Ka}_{3}+k_{3f}\cdot{Ka}_{7})\cdot{Ka}_{8}+$  $\left[ H \right]^{2}\cdot{Ka}_{6}\cdot(k_{4f}\cdot k_{5f}\cdot{Ka}_{8}+k_{3f}\cdot{Ka}_{4}\cdot(k_{4f}+(k_{4r}+k_{5f})\cdot{Ka}_{8})))\cdot K_{i}\cdot K_{ii}$ |
| $d_{1}$ | $\left[ H \right]^{2}\cdot k_{2f}\cdot k_{3f}\cdot k_{4f}\cdot{Ka}_{2}\cdot{Ka}_{4}\cdot{Ka}_{6}\cdot{Ka}_{8}\cdot K_{ii}$ |
| $d_{2}$ | $\left[ H \right]^{2}\cdot k_{4f}\cdot(k_{1f}\cdot k_{3f}+k_{2r}\cdot k_{5f})\cdot{Ka}_{2}\cdot{Ka}_{4}\cdot{Ka}_{6}\cdot{Ka}_{8}\cdot K_{i}$ |
| $d_{3}$ | $k_{1f}\cdot k_{2f}\cdot k_{4f}\cdot{Ka}_{2}\cdot{Ka}_{3}\cdot{Ka}_{4}\cdot{Ka}_{6}+\left[ H \right]^{2}\cdot{Ka}_{2}\cdot(k_{1f}\cdot k_{2f}\cdot k_{4f}+k_{1f}\cdot k_{3f}(k_{2f}+k_{4f})\cdot{Ka}_{4}+$  $k_{2r}\cdot k_{4f}\cdot k_{5f}\cdot{Ka}_{4})\cdot{Ka}_{6}+\left[ H \right]\cdot{Ka}_{2}\cdot{Ka}_{4}\cdot(k_{2r}\cdot k_{4f}\cdot k_{5f}\cdot{Ka}_{1}+k_{1f}\cdot{(k}_{2f}\cdot k_{4f}+$  $k_{3f}\cdot k_{4f}\cdot{Ka}_{1}+k_{2f}\cdot k_{3f}\cdot{Ka}_{5})) \cdot{Ka}_{6}+\left[ H \right]^{3}\cdot{Ka}_{4}(k_{2r}\cdot k_{4f}\cdot k_{5f}\cdot{Ka}_{6}+k_{1f}\cdot k_{3f}\cdot(k_{2f}\cdot{Ka}_{2}+$  $k_{4f}\cdot{Ka}_{6})))\cdot{Ka}_{8}\cdot K_{i}\cdot K_{ii}$ |
| $d_{4}$ | $\left[ H \right]\cdot k_{1f}\cdot k_{2r}\cdot k_{4f}\cdot{Ka}_{2}\cdot{Ka}_{4}\cdot{Ka}_{6}\cdot{Ka}_{8}\cdot K_{i}$ |
| $d_{5}$ | $k_{1f}\cdot k_{2r}\cdot k_{4f}\cdot(\left[ H \right]^{2}+(\left[ H \right]+{Ka}_{1})\cdot{Ka}_{2})\cdot{Ka}_{4}\cdot{Ka}_{6}\cdot{Ka}_{8}\cdot K_{i}\cdot K_{ii}$ |

Table S2B: Meaning of *n_i_* and *d_i_* coefficients for Models II in Figure 5. Where the Equation for Model II is:

$$\frac{n_{0}+ n_{1}[K]}{d_{0}+ d_{1}\left[ Inh \right]+ d_{2} \left[ Inh \right]\left[ K \right]+ d_{3}{\left[ K \right]+ d_{4} \left[ Inh \right][K]}^{2}+ d_{5} {[K]}^{2}}$$

**Table S2C**

Table S2C: Model III

| Parameter | Definition |
| --- | --- |
| $n_{0}$ | $\left[ E_{t} \right]\cdot\left[ H \right]^{3}\cdot k_{2f}\cdot k_{3f}\cdot k_{4f}\cdot k_{5f}\cdot{Ka}_{2}\cdot{Ka}_{4}\cdot{Ka}_{6}\cdot{Ka}_{8}\cdot K_{i}\cdot K_{ii}$ |
| $n_{1}$ | $\left[ E_{t} \right]\cdot\left[ H \right]^{2}\cdot k_{1f}\cdot k_{2f}\cdot k_{3f}\cdot k_{4f}\cdot{Ka}_{2}\cdot{Ka}_{4}\cdot{Ka}_{6}\cdot{Ka}_{8}\cdot K_{i}\cdot K_{ii}$ |
| $d_{0}$ | $\left[ H \right]\cdot k_{2f}\cdot{Ka}_{2}\cdot(\left[ H \right]^{3}\cdot k_{3f}\cdot(k_{4r}+{k_{5f})\cdot Ka}_{4}\cdot{Ka}_{8}+\left[ H \right]\cdot{Ka}_{4}\cdot(k_{4f}\cdot(k_{3f}+k_{5f})+$  $k_{3f}(k_{4r}+k_{5f})\cdot{Ka}_{5})\cdot{Ka}_{6}\cdot{Ka}_{8}+k_{4f}{\cdot Ka}_{4}\cdot{Ka}_{6}\cdot(k_{5f}\cdot{Ka}_{3}+k_{3f}\cdot{Ka}_{7})\cdot{Ka}_{8}+$  $\left[ H \right]^{2}\cdot{Ka}_{6}\cdot(k_{4f}\cdot k_{5f}\cdot{Ka}_{8}+k_{3f}\cdot{Ka}_{4}\cdot(k_{4f}+(k_{4r}+k_{5f})\cdot{Ka}_{8})))\cdot K_{i}\cdot K_{ii}$ |
| $d_{1}$ | $\left[ H \right]^{2}\cdot k_{2f}\cdot k_{4f}\cdot{Ka}_{2}\cdot{Ka}_{4}\cdot{Ka}_{6}\cdot{Ka}_{8}\cdot{(k_{5f}\cdot K}_{i}+k_{3f}\cdot K_{ii})$ |
| $d_{2}$ | $\left[ H \right]\cdot k_{1f}\cdot k_{2f}\cdot k_{4f}\cdot{Ka}_{2}\cdot{Ka}_{4}\cdot{Ka}_{6}\cdot{Ka}_{8}\cdot K_{i}$ |
| $d_{3}$ | $k_{1f}\cdot k_{2f}\cdot k_{4f}\cdot{Ka}_{2}\cdot{Ka}_{3}\cdot{Ka}_{4}\cdot{Ka}_{6}+\left[ H \right]^{2}\cdot{Ka}_{2}\cdot(k_{1f}\cdot k_{2f}\cdot k_{4f}+k_{1f}\cdot k_{3f}(k_{2f}+k_{4f})\cdot{Ka}_{4}+$  $k_{2r}\cdot k_{4f}\cdot k_{5f}\cdot{Ka}_{4})\cdot{Ka}_{6}+\left[ H \right]\cdot{Ka}_{2}\cdot{Ka}_{4}\cdot(k_{2r}\cdot k_{4f}\cdot k_{5f}\cdot{Ka}_{1}+k_{1f}\cdot{(k}_{2f}\cdot k_{4f}+$  $k_{3f}\cdot k_{4f}\cdot{Ka}_{1}+k_{2f}\cdot k_{3f}\cdot{Ka}_{5})) \cdot{Ka}_{6}+\left[ H \right]^{3}\cdot{Ka}_{4}(k_{2r}\cdot k_{4f}\cdot k_{5f}\cdot{Ka}_{6}+k_{1f}\cdot k_{3f}\cdot(k_{2f}\cdot{Ka}_{2}+$  $k_{4f}\cdot{Ka}_{6})))\cdot{Ka}_{8}\cdot K_{i}\cdot K_{ii}$ |
| $d_{4}$ | $k_{1f}\cdot k_{2r}\cdot k_{4f}\cdot(\left[ H \right]^{2}+(\left[ H \right]+{Ka}_{1})\cdot{Ka}_{2})\cdot{Ka}_{4}\cdot{Ka}_{6}\cdot{Ka}_{8}\cdot K_{i}\cdot K_{ii}$ |

Table S2C: Meaning of *n_i_* and *d_i_* coefficients for Models III in Figure 5. Where the Equation for Model III is:

$$\frac{n_{0}+ n_{1}[K]}{d_{0}+ d_{1}\left[ Inh \right]+ d_{2} \left[ Inh \right]\left[ K \right]+ d_{3}{\left[ K \right]+ d_{4} [K]}^{2}}$$
